# Degradation of a poly(3-hydroxybutyrate-*co*-3-hydroxyvalerate) (PHBV) compound in different environments^☆^

**DOI:** 10.1016/j.heliyon.2024.e24770

**Published:** 2024-01-24

**Authors:** Pavlo Lyshtva, Viktoria Voronova, Jelena Barbir, Walter Leal Filho, Silja Denise Kröger, Gesine Witt, Lukas Miksch, Reinhard Sabowski, Lars Gutow, Carina Frank, Anita Emmerstorfer-Augustin, Sarai Agustin-Salazar, Pierfrancesco Cerruti, Gabriella Santagata, Paola Stagnaro, Cristina D'Arrigo, Maurizio Vignolo, Anna-Sara Krång, Emma Strömberg, Liisa Lehtinen, Ville Annunen

**Affiliations:** a Tallinn University of Technology, Ehitajate tee 5, 19086, Tallinn, Estonia; b Hamburg University of Applied Sciences, Ulmenliet 20, 21033, Hamburg, Germany; c Alfred Wegener Institute, Am Handelshafen 12, 27570, Bremerhaven, Germany; d Austrian Centre of Industrial Biotechnology, Krenngasse 37/2, A-8010, Graz, Austria; e Institute for Polymers, Composites and Biomaterials, National Research Council, Via Campi Flegrei 34, 80078, Pozzuoli (NA), Italy; f Institute of Chemical Sciences and Technologies "Giulio Natta", National Research Council, Via De Marini 6, 16149, Genova, Italy; g IVL Swedish Environmental Research Institute, Valhallavägen 81, 114 28, Stockholm, Sweden; h Turku University of Applied Sciences, Joukahaisenkatu 3, 20520, Turku, Finland

**Keywords:** PHBV, Polymer blends, Degradation, Natural environment, Laboratory scale testing, Morphological properties

## Abstract

Poly(3-hydroxybutyrate-*co*-3-hydroxyvalerate) (PHBV) is a promising biodegradable bio-based material, which is designed for a vast range of applications, depending on its composite. This study aims to assess the degradability of **a** PHBV-based compound under different conditions. The research group followed different methodological approaches and assessed visual and mass changes, mechanical and morphological properties, spectroscopic and structural characterisation, along with thermal behaviour. The Ph-Stat (enzymatic degradation) test and total dry solids (TDS)/total volatile solids (TVS) measurements were carried out. Finally, the team experimentally evaluated the amount of methane and carbon dioxide produced, i.e., the degree of biodegradation under aerobic conditions. According to the results, different types of tests have shown differing effects of environmental conditions on material degradation. In conclusion, this paper provides a summary of the investigations regarding the degradation behaviour of the PHBV-based compound under varying environmental factors.

The main strengths of the study lie in its multi-faceted approach, combining assessments of PHBV-based compound degradability under different conditions using various analytical tools, such as visual and mass changes, mechanical and morphological properties, spectroscopic and structural characterization, and thermal behavior. These methods collectively contribute to the robustness and reliability of the undertaken work.

## 1 Introduction

Plastics are used widely, and the numerous advantages of plastics have made them an unavoidable and vital part of everyday human life [1]. Geyer et al. [2] described this dependency of humans on plastics as “a world without plastics, or synthetic organic polymers, seems unimaginable today”. However, plastics started to pose a significant threat to the environment and human health, and the diverse problems caused by the accumulation and disintegration of the exceedingly growing plastic waste in the oceans, inland waters and terrestrial environments is becoming more evident every day [3].

Considering the fact that the human population is constantly growing, the usage of plastics is increasing globally, while efficient plastics disposal and recycling is still limited in many areas across the world. This trend has provoked the urgent need to reduce, reuse and recycle the plastics currently used and produced on one hand, but also to strongly focus on the new more sustainable solutions to replace fossil-based plastics.

One of the most promising solutions is found in biodegradable and bio-based plastics, commonly called “bioplastics”. At present, these innovative materials are an important part of the bioeconomy, creating 400 million euros in annual turnover and providing 1.5 million jobs within the EU [4]. Although bioplastics may considerably increase resource efficiency, especially if bio-based materials are either reused or recycled, they still constitute a small portion of the plastics industry.

Biodegradation is an important aspect of more sustainable bio-based plastics. Acknowledging this, the biodegradability in the marine environment of bio-based plastics (referred to as biodegradable and/or compostable in industrial composting conditions) is difficult to predict. Indeed, the ability to biodegrade can vary a lot depending on the specific item and the environmental conditions of the ecosystem of interest [5].

Among a range of biodegradable and bio-based plastics, polyhydroxyalkanoates (PHAs) are considered an alternative to conventional fossil-based plastics. PHAs are a family of bio-based, biodegradable and non-toxic polymers with thermoplastic properties [[6], [7], [8]], which are naturally produced by bacteria [9]. Poly(hydroxybutyrate-*co*-hydroxyvalerate) (PHBV) is one of the examples of the PHA family that can be produced from renewable vegetable resources [[10], [11], [12]]. PHBV is classified as recyclable, industrially compostable and suitable for injection moulding applications. Mechanical recycling of the material can be carried out up to six times, depending on the proportion of HV (3-hydroxyvalerate) content in the polymer [13]. According to the European Bioplastics update in 2021, the capacity of global production of bioplastics continues to increase and diversify. After poly(lactic acid) (PLA) and poly(butylene adipate-*co*-terephthalate) (PBAT) with 18.9 % and 19.2 % respectively, PHA production is about 1.8 % and expected to grow to 6.4 % by 2026.

According to previous research, PHBV bio-polyester degrades naturally in different environments. Enzymatic degradation of PHBV was studied on a molecular basis by some authors [14,15]. For such reactions, enzymes are added as biocatalysts. Similarly, the degradation in soil by fungi [16] and in seawater and compost [17] was investigated. In natural environments, the weight loss of PHBV compounds reaches 60 % after 6 weeks for seawater media [17] and the degree of biodegradation over 70 % after 18 weeks for soil media [18]. Thus, PHBV's notable properties made the material become an upcoming applicant for vital applications such as packaging, medicine and auto manufacturing [8]. Nonetheless, the limitations of PHBV usage are caused by its brittleness, poor mechanical properties [19,20] and the high price - on average of 5 €/kg [6].

In this context, the purpose of this study is to evaluate the degradation of PHBV-based material that is designed for beach toys and fishing bait applications, due to which the material in this work is named T-PHBV, where “T” refers to its function. Since plastic debris can appear in various forms in the environment, ranging from solid pieces to fragmented microplastics, and this study aims to account for all potential shapes.

For on-field tests conducted in seawater, dog-bone shaped specimens were chosen to represent objects like shovels and rakes. These dog-bone samples were designed to be securely attached to a rack for immersion in seawater for extended periods. Uni-axial tensile tests were directly performed on these samples, while other properties were monitored either from their surface or by cutting pieces from them.

To facilitate quicker results and tangible changes in physical properties, film samples were utilized for degradation tests in the climatic chamber and soil conditions.

The study aims to assess the degradation of T-PHBV material in both natural environments and controlled laboratory settings. The provided polymer is a blend, containing less than 15 % of additives, primarily impact modifiers, to suit its designated applications.

## 2 Materials and Methods

### 2.1 Material description

The set of degradation tests was performed on T-PHBV-based material, supplied by NaturePlast (NPL, Rue François Arago, France). The material is a thermoplastic resin of PHBV Poly(3-hydroxybutyrate-*co*-3-hydroxyvalerate) with approximately 5 wt% 3-hydroxyvalerate units produced from annually renewable vegetal resources. The test samples were supplied (or purposely prepared) in different shapes: dog-bone bars (Dumbbell 1A - Overall length 150 mm, dimension of test area 80 mm × 10 mm, distance between shoulders 28 mm, thickness 4 mm), granules (2.5 × 3 mm^2^; 3 × 3 mm^2^), sheets (12 × 12 cm^2^, thickness ca. 0.2 mm), film (thickness 350 μm) and microparticles (<200 μm size), depending on the test they are designed to. The supplied PHBV has a melting temperature of 170–176 °C, Young's modulus 3300 MPa, tensile maximal strength and tensile strength at break 38 MPa, tensile elongation at break 3.7 %.

On-field degradation tests were carried out on T-PHBV samples exposed to various natural environments (soil, home compost, fresh water, as well as in the Mediterranean and coastal North Sea seawater) for up to 1 year. Laboratory scale tests include the degradation evaluation in a climatic chamber, enzymatic degradation, biodegradability determination under controlled aerobic conditions and degradation under anaerobic conditions. Besides the decay of the mechanical properties (evaluated by uni-axial tensile and Charpy tests), changes in the samples' aspect and weight as well as in their thermal behaviour (TGA and DSC), molecular (GPC), spectroscopic (ATR-FTIR), morphological (SEM), pH Stat, carbon dioxide (CO_2_) evolution and methane (CH_4_) production features were also investigated.

### 2.2 Degradation of T-PHBV plastic in the natural environment

#### 2.2.1 Degradation in soil

Dumbbell-shaped T-PHBV specimens, previously conditioned in a climatic chamber at 25 °C and 50 % RH, were buried 5 cm deep in the soil. In order to standardise the biodegradation tests, commercial garden soil (a mixture of peats and composted vegetal materials) (pH 6.5, dry apparent density 220 kg m^−3^, total porosity 85 % VV^−1^) was used. The soil, placed in a pot (60 cm × 40 cm), was kept in the laboratory at room temperature and at a constant RH of 50 % by regular watering for optimal microbial activity. The specimens were periodically collected from the soil, cleaned from dirt residues with a brush and vacuum dried at 40 °C for 24 h before testing. Tensile tests were performed using an Instron model 5564 dynamometer (U.S.A.) equipped with a 1 kN load cell. The test was performed on six specimens at 23 ± 2 °C, 45 ± 5 % RH with a 5 mm min^−1^ clamp separation rate.

A FEI Quanta 200 FEG (Eindhoven, The Netherlands) Scanning Electron Microscope (SEM) was used to investigate the surface morphology of the buried films. In order to remove dirt traces that could affect the analysis, all the buried samples were cleaned by sonication in a water bath and gently dried with blotting paper. Previously, SEM observation films were coat-sputtered with a gold/palladium alloy.

Gel Permeation Chromatography (GPC) was carried out with the samples before and after the soil burial test. A Malvern-Viscotek GPC max chromatographic system was used, equipped with a TDA 305 Tetra Detector system, pre-column and two columns Phenogel Phenomenex with an exclusion limit of 106 and 103 Da. A universal calibration curve was obtained by using 12 polystyrene standards with narrow polydispersity and a MW range of 0.5–3000 kDa. An isocratic elution of chloroform at a flow rate of 1 mL min^−1^ was applied.

#### 2.2.2 Degradation in home compost

Home compost experiments were performed in a drum composter with a volume of 70 L (PP Drum composter two chambers, UPP Products GmbH) under natural conditions following UNE EN ISO 20200 (2016) with modifications, since ISO 20200:2015 is a standard for composting of biodegradable plastics under laboratory conditions. Therefore, compost material was adjusted to a more realistic mixture containing: 35.7 wt% mature compost, 22.3 wt% potatoes (1 × 1 cm^2^ pieces), 16.7 wt% wood shavings, 15.0 wt% domestic compost waste, 7.4 wt% fresh grass and 3 wt% rabbit dung. In addition, ISO 20200:2015 requires a controlled ambient temperature of 40 ± 2 °C; however, in the home compost experiment a mean ambient temperature of 15.1 °C (min. 4.2 °C, max. 26.5 °C) was reached under field conditions. The temperature was recorded by a sensor (HOBO Pendant® UA-001-64) every 6 h for the entire duration of the experiments. During the test period, the mean temperature in the compost was 18.6 °C. with min. 7.5 °C and max. 39.1 °C. For sample preparation, T-PHBV films were cut into a 5 × 5 cm^2^ piece and placed into a stainless-steel cage with a mesh size of 2 mm, in accordance with Arrieta et al. [21]. Degradation experiments were performed for eight weeks, analysed and documented visually once per week. After eight weeks of exposure, the T-PHBV cage was taken out of the drum composter. Subsequently, the cage was removed and the T-PHBV film piece was cleaned carefully with ultrapure water and lint-free tissue for further investigations.

PE was used as a negative control sample in the home compost experiments, and intriguingly, no degradation of PE was observed throughout the entire duration of the study.

#### 2.2.3 Degradation in freshwater

T-PHBV film preparation for freshwater experiments was performed in the same way as for the home-compost experiments. For investigating the degradation of T-PHBV in freshwater, the cage containing the T-PHBV film was exposed to the tidal freshwater of Elbe River at the measuring station “Seemannshöft” (Hamburg Institute for Hygiene and Environment), which is 2 km downstream from the Hamburg harbour (53°31′60″ N; 9°52′60″ E). To prevent the introduction of microplastics into the river Elbe, the stainless-steel cage containing T-PHBV film was placed into a flow-through chamber in which the suspended material from the water was collected. During exposure of T-PHBV film in the flow-through chamber, the mean temperature was 18.0 °C (minimum 11.3 °C; maximum 22.8 °C) and the pH was 7.7 ± 0.2. After 12 weeks of exposure, the cage was removed from the flow-through chamber (which was full of suspended material). The T-PHBV film was removed from the cage and carefully cleaned with ultrapure water and lint-free tissue. Afterwards, further investigations of the T-PHBV film's visual and mass changes were carried out. In addition, the thermal behaviour of the T-PHBV film was investigated by differential scanning calorimetry (DSC) and the morphological properties by SEM.

In the Elbe experiments, silicone (PDMS) was utilized as a biocompatible control. Interestingly, no degradation of PDMS was observed during the course of the experiment.

#### 2.2.4 Degradation in estuarine water and estuarine mud

Four dumbbell-shaped bars of the T-PHBV materials were incubated for six months in natural seawater in a recirculating flow-through system (0.6 × 0.4 × 0.4 m^3^; length, width, height, respectively) with a total volume of 160 L. The bars were fixed and tightened with nylon cords between two racks, to avoid contact between the bars and between the bars and the tank. The system was placed in a temperature-controlled room at 15 °C and a light/dark cycle of 12:12 h. The salinity of the water was monitored with a refractometer (Atago, Tokyo, Japan) every two weeks. Due to evaporation, the salinity increased slightly but continuously over time. If the salinity exceeded 38 ppt, it wasadjusted to 35 ppt by adding deionized water. The redox potential, temperature, and pH of the seawater were monitored every two weeks with a pH meter pH 3110 (Xylem Analytics, Weilheim, Germany) connected to a SenTix® 41 pH (Xylem Analytics, Weilheim, Germany) or a double pore redox (Hamilton AG, Waengi, Switzerland) electrode. Ammonium nitrite and nitrate were measured with an automated analyser (QuAAtro39 AutoAnalyzer, SEAL Analytical GmbH, Germany) once every month.

Four dumbbell-shaped T-PHBV-bars were incubated for six months in natural untreated mud from the Weser estuary (53°32′21.3"N 8°34′33.2"E). The mud was evenly distributed in a box (0.55 × 0.35 × 0.3 m^3^, length, width, and height respectively) to form a layer of 15 cm. The plastic bars were placed on top of the layer with sufficient distance between each other. Subsequently, the bars were covered by another layer of the mud of about 10 cm. To prevent the mud from drying, about 1 L of deionized water was sprayed on the mud surface every 2 weeks. Redox potential, temperature and pH were monitored every week.

The bars were removed from the seawater and the mud after 6 months and carefully rinsed with deionized water, dried and stored separately in sealed freezer bags. Samples of approximately 1 cm^2^ were cut out from the bars and the surfaces were sputtered with gold/palladium. SEM micrographs of the surfaces were taken with a FEI Quanta 200 microscope.

#### 2.2.5 Degradation in seawater

**Fig. 1:**
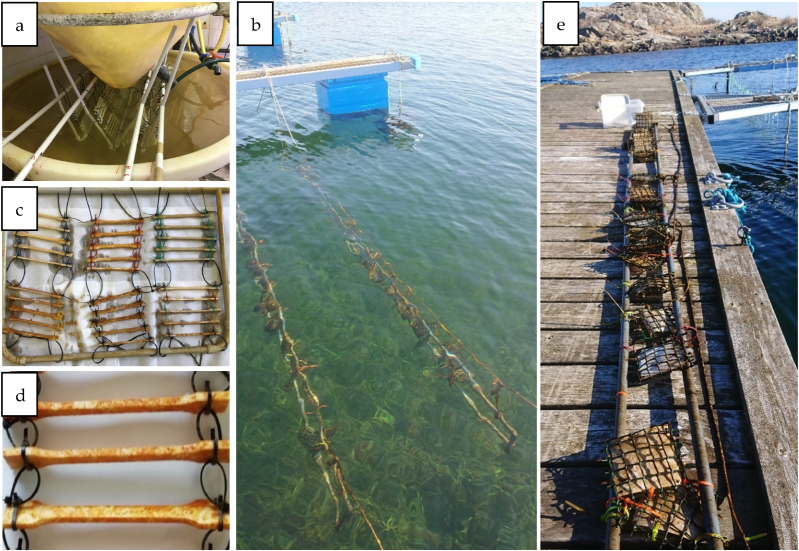
Set up for field exposure of T-PHBV (a) Dumbbell bars immersed in tanks with circulating Mediterranean Sea water and (b) sheets submerged at 10–20 cm depth in coastal, surface seawater in the North Sea. Bars (c, d) and sheets (e) visual aspect after 12-months of exposure.

Degradation tests in the Mediterranean seawater were carried out on T-PHBV bars (Dumbbell 1A provided by NPL) suitable for uni-axial tensile tests. The bars were tied to frames and immersed in tanks with circulating seawater in mid-October 2020 at the CNR Sea-Lab Station in Camogli (Genova, Italy). For the degradation tests in the North Sea, T-PHBV sheets were placed in individual loose net pockets and submerged in a coastal, estuarine area at a depth of ca. 10–20 cm in mid-December 2020 (Fig. 1). Measurements were done on unexposed samples and after 1- (only for the Mediterranean Sea), 3-, 6-, and 12-months exposure (T0, T1, T3, T6, T12 respectively). Temperature and other seawater parameters were monitored during the period of interest. For the Mediterranean seawater, temperature varied from a minimum of 11 °C to a maximum of 26 °C; pH varied randomly in the range 8.1–8.4; PSU in the range 36.3–42.9 g/kg; dissolved oxygen 44–51 %. For the North Sea samples, the water temperature varied between −1 °C and +24 °C and salinity ranged from 15 to 32 PSU over the exposure period, which involved both seasons of ice cover and intense summer light. Uni-axial tensile tests on 5/6 replicates (Dumbbell 1A bars or 10 × 80 mm strips cut from the sheets) were carried out with a Shimadzu AG-X plus universal testing machine equipped with a 10 kN load cell (EN ISO 527-1,-3). ATR-FTIR analysis was performed operating with a PerkinElmer Spectrum TwoTM spectrometer equipped with a diamond crystal. At least 5 spectra (4000–400 cm^−1^ range) were collected from different sample points, averaged and normalised, after correction of the spectral baseline, with respect to the band at around 1380 cm^−1^, assigned to the –CH_3_ symmetric wagging after correction of the spectral baseline, as the suitable internal standard for T-PHBV infrared analysis [11]. TGA measurements were carried out with a PerkinElmer TGA 8000 analyser on specimens of 10 mg (cut from the bars/sheets), heating at a rate of 10 °C/min under N_2_ from 40 to 700 °C, then introducing pure O_2_ at the rate of 20 °C/min up to 850 °C (EN ISO 11358-1). Calorimetric analysis was performed with a DSC Mettler 821^e^ instrument on specimens of 10 mg (cut from the bars/sheets), applying a heating-cooling-heating cycle in the range 0 ÷ 210 °C, under N_2_ and at a scan rate of 20 °C/min (EN ISO 11357-1-3). Morphological observations were done with a Hitachi TM3000 bench top SEM microscope operating at 15 kV. Prior to observation, sample surfaces were sputtered with silver.

Thermal behaviour was analysed by DSC (Netsch DSC 214 Polyma, Mettler-Toledo). Therefore, the glass transition and melting point of the T-PHBV material were analysed before and after degradation experiments. Afterwards, the samples were heated in two cycles from 20 °C to a maximum temperature of 250 °C with a heating rate of 10 °C/min under an inert nitrogen atmosphere.

### 2.3 Degradation under controlled conditions at a laboratory scale

#### 2.3.1 Degradation in a climate chamber

The studied material was degraded in climatic chamber in order to follow the effect of UV irradiation on the physical properties of material. Accelerated weathering of dumbbell-shaped specimens (see Section 2.2.1) was carried out at 40 °C and 50 % RH in an Angelantoni SU250 (Italy) climatic chamber equipped with a mercury UV lamp (λ > 250 nm). Aged specimens were periodically collected and tested to assess variations in molecular weight and tensile properties, as described in Section 2.2.1.

#### 2.3.2 Enzymatic degradation

The enzymatic degradability of T-PHBV with a PHBV depolymerase under environmentally relevant seawater conditions was determined by pH Stat titration [22]. A PHBV depolymerase (accession number: P52090) gene from *Paucimonas lemoignei* (formerly *Pseudomonas lemoignei)* was codon optimised, cloned and expressed as a C-terminally His_6_-tagged fusion in *E. coli*. Recombinant PHBV depolymerase was purified by metal affinity chromatography using an ÄKTA pure protein purification system.

The PHBV depolymerase of *Pseudomonas lemoignei* plays a crucial role in degrading PHBV in its environment [23]. To assess its thermal properties, it is necessary to conduct measurements under controlled conditions using purified enzymes within a laboratory setting.

T-PHBV granules were ground with a cryogenic mill (SPEX SamplePrep, 6775 Freezer/Mill) and subsequently sieved to obtain microparticles <200 μm. For the titration assay, 30 mg of T-PHBV microplastics were suspended in 10 mL artificial seawater with 35 ppt salinity. The automatic titration system TitroLine 7000® from SI Analytics GmbH (Mainz, Germany) was used for all measurements. Briefly, the pH in the T-PHBV suspensions was continuously monitored by the titration system and kept constant at pH 8.2 by the addition of 10 mmol L^−1^ NaOH-solution. 5 μL of PHBV depolymerase (14.7 mg protein/ml) was added to the suspension. During the hydrolysis of T-PHBV, carboxyl groups were formed, leading to a decrease in the pH. The amount of NaOH solution needed to keep the pH of the suspension constant was measured for 60 min, from which the hydrolysis rate of the T-PHBV material was calculated. An enzyme-free microplastic blank was run immediately before the start of each measurement as reported in detail by Miksch et al. (2021). The blank was subtracted from the hydrolysis rate of the microplastic by the enzyme. The measurements were performed at 5, 15 and 25 °C in a thermostat jacket to maintain a constant temperature in the reaction vial [22]. The temperature in the jacket was controlled with a circulation thermostat (Lauda, Lauda-Königshofen, Germany). Enzyme blanks without microplastics were measured at each temperature. Routine measurements were carried out in triplicate. The electrode of the titration system was calibrated every day before use.

As control, identical T-PHBV bars have been stored simultaneously to the exposure experiment under dry and dark conditions in the laboratory.

Statistical analysis and graphs were done with the program GraphPad Prism version 7.05 for Windows, GraphPad Software, La Jolla California USA, https://www.graphpad.com. The data for maximum tensile strength were compared by a 1-factorial ANOVA with the subsequent Tukey's HSD test for multiple comparisons. The significance level of all statistical analyses was α = 0.05. The temperature dependency of the hydrolysis rate of T-PHBV was described by an exponential model (Equation (1)):

(1) $$f_{\left(x\right)}=a\;\cdot \;e^{bx}$$

#### 2.3.3 Composting under controlled aerobic conditions

Degradation tests of PHBV-based material in granules were performed under controlled aerobic conditions following EVS-EN ISO 14855–1:2012. Sludge obtained from Keila WWTP (Harjumaa, Estonia) was used as inoculum instead of compost. Before the beginning of the testing, the inoculum was prepared by treating it from large inert objects such as glass, stones and wood, sieved on 1 × 1 cm mesh and had the succeeding characteristics: pH – 7.1, TS – 50 % TVS – 46 %, TOC – 49 % (Apparatus: Vario TOC, Solids Module; 950 °C, Elementar GmbH, Germany), Total nitrogen (total N) – 34000 mg/kg KA (Kjeldahl mod, in accordance to ISO 11261:1995). Also, supplementary characteristics of the plastic were determined, including TS – 99.65 %, TVS – 98.82 %, and TOC – 57 %.

Based on EVS-EN ISO 14855–1:2012, the test plastic material was mixed with the inoculum in a ratio of 1:6 (dry mass to dry mass) and placed in a stationary composting vessel in the dark light, where the mixture was actively aerated via carbon dioxide-free air under optimal conditions of moisture content (50 ± 5 %), oxygen concentration (>6 %), temperature (58 ± 2 °C) and pH (from 7.0 to 9.0) for 6 months. Altogether, nine vessels were used, where three vessels were used for the inoculum (blank), three vessels for the test material, and three vessels for the control sample (TLC grade cellulose with a particle of less than 20 μm).

The carbon dioxide produced was constantly monitored and measured at regular intervals in all composting vessels. The amount of CO_2_ produced from the combination of test material and inoculum in a composting vessel was compared to the amount of CO_2_ produced only from inoculum in a composting vessel (blank sample). Additionally, weekly measurements consisted of adjusting the humidity and shaking the composting vessels, and pH measurements were taken to maintain a stable composting process. The titration method based on EVS-EN ISO 19679:2020 was chosen for the evaluation of carbon dioxide produced by blank (CO_2_)_B_ and test (CO_2_)_T_ vessels in g/vessel, from which the percentage of biodegradation D_t_ of materials can be found (Equation (2)):

(2) $$D_{t}=\frac{{\left({CO}_{2}\right)}_{T}-{\left({CO}_{2}\right)}_{B}}{{ThCO}_{2}}\cdot 100\%$$

In pursuance of EVS-EN 13432:2003, the percentage of T-PHBV material biodegradation was indicated as the maximum degradation of the reference material after a plateau phase was reached for both T-PHBV and TLC grade cellulose.

Final measurements included the comparison of morphological properties (SEM) before and after the aerobic degradation testing.

#### 2.3.4 Degradation under controlled anaerobic conditions

The degradation of PHBV-based plastic was tested by a methane production potential test in anaerobic conditions in a laboratory. The device used was AMPTS II Automatic Methane Potential Test System produced by Bioprocess Control Sweden AB. The customised test is based on standard ISO 14853:2016 Plastics. Determination of the ultimate anaerobic biodegradation of plastic materials in an aqueous system. The method of measurement of biogas production.

The test material was put into inoculum in two parallel test chambers of 500 ml that were stirred in continuous cycles. The biogas formed in the chamber was led through a bottle filled with 3 M NaOH that captured CO_2_ away from the biogas, thus only methane passed to the gas detector that measured the methane gas formed during the test. A Dumbbell 1A test bar was chosen as a test sample as it was considered to represent the application (a toy) the best from its size and wall thickness. One and a half Dumbbells were put into the test chamber. The whole Dumbbell 1A test bar was only partially immersed in the inoculum; the half of a dumbbell was completely immersed. The inoculum used was from a working biogas plant in Rusko, Finland.

The amount of methane produced from the combination of test material and inoculum in a chamber was compared to the amount of methane produced only from inoculum in a chamber (0-sample). The process was active at 38 °C (Mesophilic biogas process). After incubation of 60 days the samples were removed from the chamber and rinsed with distilled water. The excess water was wiped with paper tissue and samples were left to dry in room temperature for analysis.

DSC analysis before and after the methane production potential test was made by PerkinElmer DSC 6000. FTIR analysis were done by Shimadzu LabSolutions IR.

## 3 Results and discussion

### 3.1 Visual and mass changes

The degradation of T-PHBV bars was found to be greater in natural estuarine mud compared to seawater under laboratory conditions. A slight decrease in mass of 1.6 ± 0.2 % was found after incubation in estuarine seawater for six months, while the bars incubated in natural mud from the Weser estuary for the same time period showed an average mass loss of 6.6 ± 0.5 %. The conditions varied between the two set ups due to the different nature of the medium.In the estaurine seawater, the mean temperature, salinity, redox potential and pH over the entire time period were 15.5 ± 0.5 °C, 35 ± 3 g L^−1^, 242 ± 49 mV and 8.0 ± 0.1 respectively. The concentration of nitrate never exceeded 4 mg L^−1^, the maximum concentration of nitrite was 0.03 mg L^−1^ and that of ammonium 0.02 mg L^−1^. In the box filled with natural estuarine mud, the mean temperature was similar at 13.9 ± 0.5 °C but the mean redox potential and pH were clearly different at −191 ± 29 mV and 7.3 ± 0.1 respectively.

**Fig. 2:**
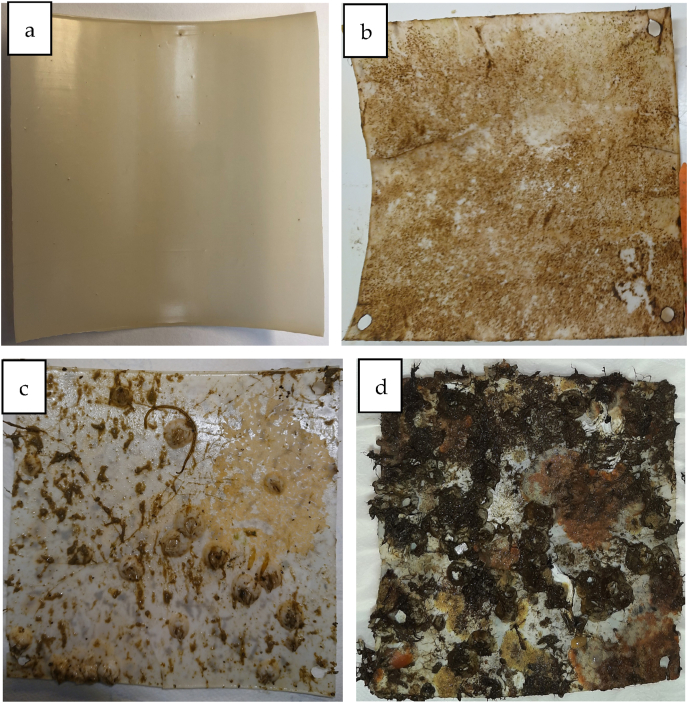
T-PHBV sheets (a) before and after field exposure in coastal, surface seawater in the North Sea. Samples submerged for 3 (b), 6 (c) and 12 (d) months show gradually increasing biofouling of marine species.

Upon 12 months of immersion in Mediterranean Sea water, no significant variation either in the visual aspect or the weight of the T-PHBV Dumbbell bars was evidenced, apart from the presence of reddish slime fouling (see Fig. 1 c, d in Section 2.2.5). For the T-PHBV sheets exposed in the open field in coastal North Sea surface water, a visual inspection demonstrated that a successive accumulation of biofouling microorganisms, algae and smaller marine animals adhered to the sample surface was observed over time (Fig. 2). After 3 months of field exposure, mainly microorganisms were present (wet weight <0.5 g), after 6 months several barnacles had adhered to the sheets (wet weight 1–8 g), and after 12 months, sheets were heavily fouled by e.g., algae, barnacles, bryozoans, polychaete tubeworm and blue mussels (wet weight 23–60 g). The great variation in biofouling between the two different sea exposure conditions is ascribable to the fact that only the North Sea sheets were exposed to open sea water and also offered a larger flat surface compared to the Mediterranean samples (Dumbbell bars in lab tanks with circulating seawater).

**Fig. 3:**
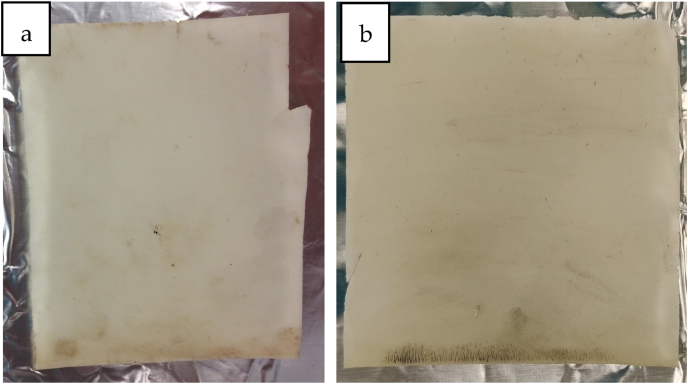
PHBV film after eight weeks degradation in home-compost (a) and after 12 weeks in river Elbe (b): dark-coloured edges with cracks and fractures were observed after degradation experiments.

The PHBV films showed slight optical changes after eight weeks of storage in a home compost (Fig. 3). During exposure in home compost, the edge of the film became discoloured; it was dark in colour after eight weeks and had cracks. In addition, fractures up to a large broken-off fragment of T-PHBV were observed (Fig. 3a). The mass of T-PHBV (≥2 mm) decreased by 8.74 % from 1337.82 g to 1215.37 g during the experiments. During degradation experiments in the river Elbe, a significant weight loss of 43.81 % of T-PHBV film was observed. Similar to the home composting samples, the T-PHBV film showed cracks and fractures at the edge (Fig. 3b) and the whole film became darker coloured. In addition, furrows were observed throughout the entire film. It is suspected that biofouling occurred at the surface of T-PHBV, since the films became darker coloured during degradation in both home compost and freshwater. In addition, it was observed that for home composting in freshwater experiments, the degradation starts at the edges of the films, which indicated fragmentation of T-PHBV to smaller particles. Since the used cages had a mesh size of 2 mm, small particles of T-PHBV (≤2 mm) were most probably washed away and could not be detected.

T-PHBV granules under aerobic composting conditions had become swollen and mouldy within six months and half of the granules had tripled in size. The material became fragile, and granules broke easily without any additional power applied. In some cases, the granules fragmented into micro-sized particles or became powder shaped. The majority of particles had become darker, without any other significant change in colour.

**Fig. 4:**
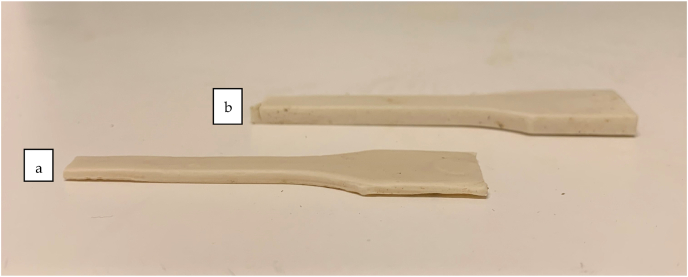
Samples 1 (a) and 2 (b) after 60 days in anaerobic conditions.

After 60 days of anaerobic degradation under controlled laboratory conditions, the visual appearance of two parallel T-PHBV samples indicated significant differences in degradation rate. Sample 1 was seemingly narrower and flatter than sample 2 (Fig. 4).

**Fig. 5:**
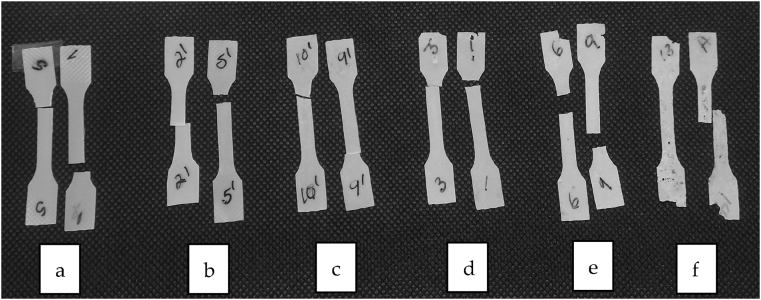
Optical pictures of the dumbbell-shaped T-PHBV samples (a) before and (b–f) during the burial test in soil for (b) 0.5 week, (c) 1 week, (d) 2 weeks, (e) 4 weeks and (f) 7 weeks.

This was also verified by measuring the mass of the two parallel samples that had been totally immersed in inoculum. The mass loss of immersed samples 1 and 2 in different chambers were 58 % and 3.3 % respectively. Despite the relatively long exposure, apart from some small stains, the colour of the samples had maintained light except. This indicated that the sludge in the chamber had not been absorbed into the polymer excessively. It also seemed that material had degraded mainly from the surface because there were no major cracks visible in the test bars. In environments like biogas plants, the degradation is mainly enzymatic. Due to relatively big particle size of enzymes, they are unable to permeate through the polymer structure. Thus, enzymatic degradation is mainly seen only at the surface [24].The dumbbell-shaped T-PHBV samples buried in soil did not exhibit substantial visual and macroscopic changes until 9 weeks of burial; however, drastic structural degradation occurred already after four weeks of soil ageing (Fig. 5). SEM micrographs revealed that the sample surface was clearly biodegraded and likely colonised by microorganisms (Fig. 13k and l). Despite the fast structural degradation of the material in the soil, no substantial mass change was observed. It is likely that, after the first weeks, the microorganisms’ proliferation on the polymer surface counterbalanced the weight loss. In this respect, accelerated ageing under photodegradative conditions did not result in visible nor mass changes, as weathering was carried out at a low temperature where no volatiles are released from the degrading sample.

As concerning the T-PHBV bars buried in soil, optical images of dumbbells periodically picked up from the soil are reported in Fig. 5. From the analysis of the micrographs, it is worth to highlight a clear fungal colonisation on the surface of the aged T-PHBV samples (Fig. 13h and i). These combined results clearly demonstrate the fast biodegradation occurring in soil.

### 3.2 Mechanical properties

Stress-strain curves obtained from uni-axial tensile tests on the T-PHBV compound showed the typical parameters of a brittle material exhibiting early rupture before yielding and necking could take place. The tensile parameters are in fairly good agreement with those given in the TDS by the NPL producer, tensile elastic modulus (E_t_) 3300 MPa; stress (σ_B_) and strain (ε_B_) at break 38 MPa and 3.7 %, respectively. The T-PHBV-based samples were mechanically tested before and during the soil burial and climate chamber ageing. Actually, with the soil-aged samples, it was possible to collect specimens to be tested only in the first two weeks (336 h), since due to prolonged permanence in soil, already after 4 weeks (672 h) a macroscopic structural deterioration with cracks and loss of integrity was observed, and the material was irreversibly shattered.

**Fig. 6:**
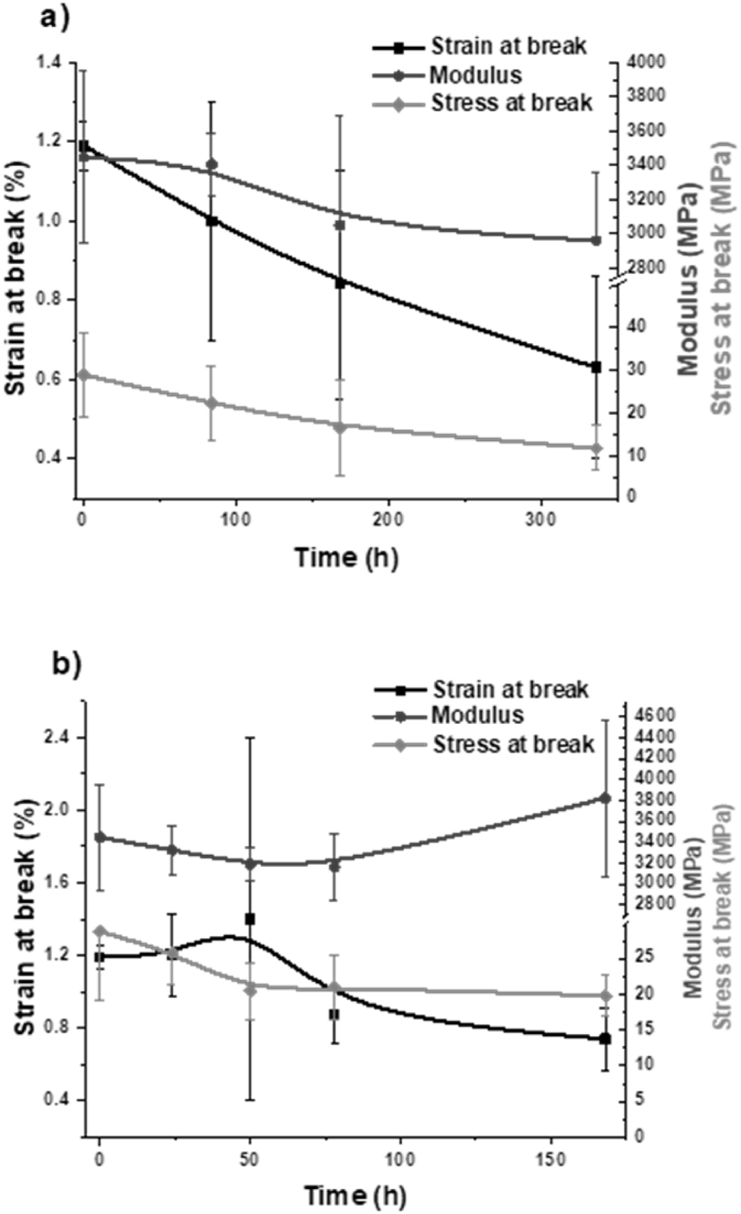
Mechanical relative ε_b_, E_t_, and σ_b_ values of T-PHBV samples aged in soil (a) and climate chamber (b).

In Fig. 6, the values of E_t_, σ_B_, and ε_B_ of both soil-buried and climate chamber-aged samples are reported. Uni-axial tensile tests evidenced that, after 2-weeks of soil burial, the strain, modulus and stress decreased by about 45 %, 15 % and 60 %, respectively (Fig. 6a). The reduction of mechanical performances could be associated with the fast T-PHBV biodegradation kinetics, as widely reported in the literature and confirmed by the quick structural collapse of T-PHBV samples after only 14 days [25,26]. Besides, T-PHBV biodegradation was supported by the peculiar soil substrate composition and microclimate generated inside the garden soil; the synergistic action of organic compost, acid pH and constant RH of 50 % could induce and hasten the biodegradation process by means of both microorganism attack (as SEM micrographs confirmed) and hydrolytic action on the polymer backbone (as demonstrated by GPC analysis) [[27], [28], [29]].

Upon immersion in Mediterranean seawater, the T-PHBV compound shows a relatively slow decrease of the mechanical properties. At T12, a reduction of the tensile elastic modulus of almost 50 % with respect to the pristine mean value (1845 vs. 3450 MPa) is accompanied by a lowering of the stress and strain at break (from 38 down to 28 MPa and from 2.6 down to 2.1 %, respectively). Analogous measurements carried out on sheet samples immersed in the North Sea retrace the behaviour described above with an even slower decrease of the mechanical properties (the tensile modulus reduces by 30 % after 1-year exposure). The different processing the sheets samples underwent (with respect to the dumbbell-shaped bars) and the lower mean temperature of the seawater they were immersed in could account for this difference.

Concerning the climate chamber results, the T-PHBV-based samples were analysed during a 7-day weathering period (Fig. 6b). Compared to the results gathered from soil-buried specimens, uniaxial tensile tests showed that mechanical performance decreased faster in the first 78 h, as strain and stress was reduced by about 30 %, while modulus dropped by 10 %. This finding is clearly related to rapid photochemical degradation, which is responsible for chain cleavage and an overall reduction in molecular weight [26,30]. With increasing weathering, the rate of property decay slowed down, as displayed by relative stress and strain changes. Interestingly, the elastic modulus increased, suggesting that chain scission was accompanied also by physical ageing and crystallisation phenomena occurring above the glass transition temperature [31].

### 3.3 Spectroscopical characterisation

**Fig. 7:**
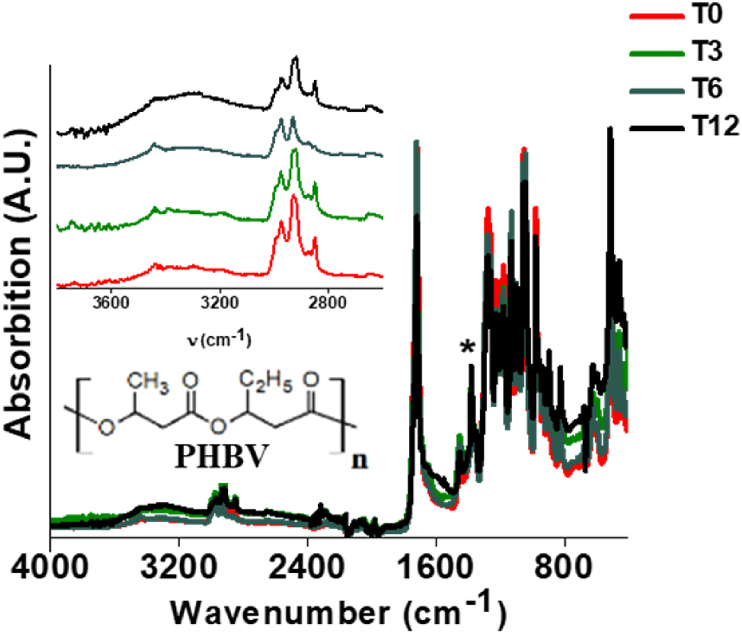
ATR-FTIR spectra recorded for T-PHBV at various times of exposure in Mediterranean seawater.

The ATR-FTIR spectrum of the pristine T-PHBV compound exhibits the vibration signals expected for a PHBV copolymer, with the C=O stretching at 1718 cm^−1^ [32]. Upon 12 months of immersion in the Mediterranean seawater, the ATR-FTIR spectra of the T-PHBV Dumbbell bars surface do not show substantial changes, except for the slow enhancement of a broad signal ranging from 3100 to 3600 cm^−1^, accompanied by a broad overtone at ca. 1600 cm^−1^ that is ascribable to the increasing presence of –OH groups formed by slow hydrolytic degradation in seawater (Fig. 7). Analogous behaviour was observed for the sheet samples immersed in the North Sea.

T-PHBV films were analysed by ATR-FTIR spectroscopy also after recovering from home compost and immersion in freshwater. A very small deviation of the spectra with respect to the pristine ones was observed in both cases. This suggests that mass loss of T-PHBV films during home compost and freshwater degradation is caused by mechanical or biological (enzymatic hydrolysis) fragmentation of the polymer chains, without substantial changes in the chemical nature of the polymer but, as said above, an increase of hydroxyl end groups.

**Fig. 8:**
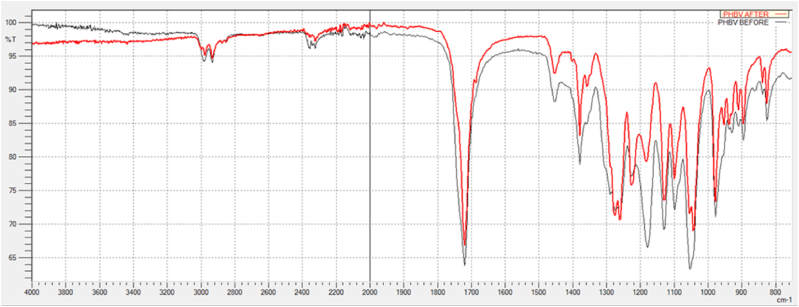
FTIR spectrums of T-PHBV before (black) and after (red) methane production potential test.

The FTIR spectra of the T-PHBV samples before and after 60 days of incubation in the chamber under anaerobic conditions (Fig. 8), showed some changes in the finger-print zone (in the wavenumber range of the C–*O*–C groups and C–O bonds).

**Fig. 9:**
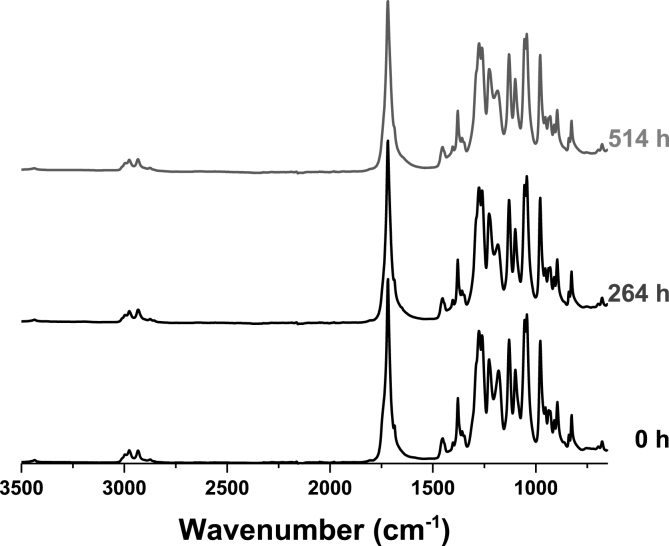
FTIR-ATR spectra during degradation in climatic chamber. Changes in FTIR-ATR spectra due to UV irradiation were followed. Characteristics spectral groups were present in the spectra without variation during the experiment.

The IR-ATR spectra of PHBV films chamber did not show any variation in the spectral groups during degradation in climatic chamber. Characterization of material after degradation in climatic chamber and in soil did not include any techniques that gives information about the degree of crystallinity of material, as it focused on macroscopic changes (Fig. 9). However, the followed protocol allowed to follow molecular changes in the sample (GPC).

### 3.4 Molecular characterisation

**Fig. 10:**
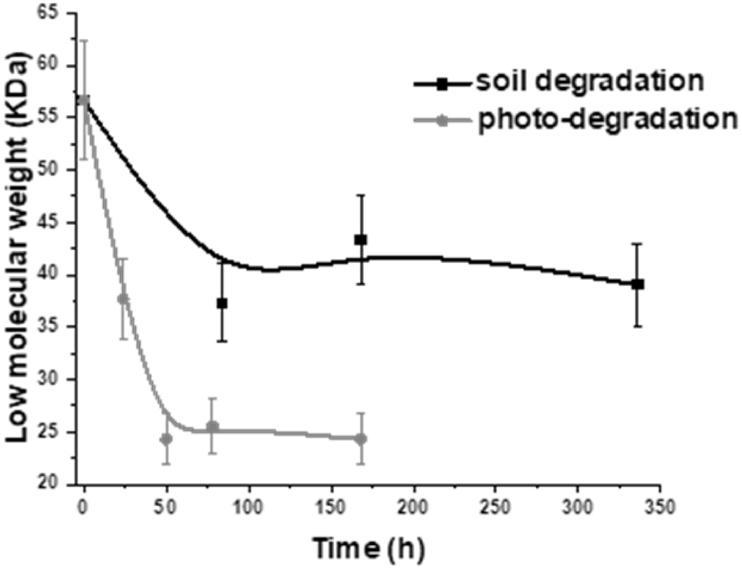
Changes in low molecular weight (Mw) of T-PHBV samples during accelerated photo-degradation in the climatic chamber and soil biodegradation.

GPC analysis was carried out on T-PHBV samples aged both in soil and in a climatic chamber (CC). The relative Mw evaluation reported in Fig. 10 evidenced a clear depolymerisation process resulting in a molecular weight decrease. More specifically, a dramatic drop in the Mw values occurred for the CC-aged samples already after 50 h ageing. This finding is clearly related to the fast photochemical degradation induced by UV lamp radiation, responsible for macromolecular chain cleavage and an overall reduction in molecular weight. Also, soil-aged samples evidenced an initial Mw decrease, likely occurring at the expense of the T-PHBV amorphous fraction depolymerisation process, followed by a quite constant weight of the polymer after two weeks. It was not possible to perform GPC analysis for longer times since the samples were no longer soluble, as their crystallinity likely increased.

GPC analysis of T-PHBV samples aged in the marine environment showed no significant changes in the molecular weight, not even in the superficial portion of the material.

### 3.5 Thermal behaviour

**Fig. 11:**
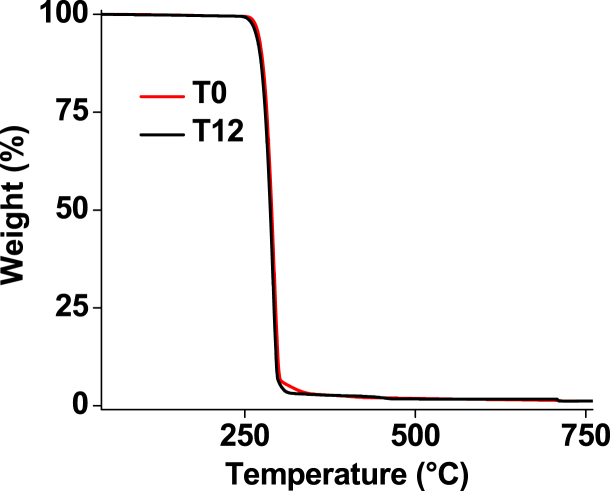
TGA curves for T-PHBV at various times of exposure in Mediterranean seawater.

Upon 1-year immersion both in the Mediterranean and the North Sea water, the thermal stability of the T-PHBV compound stayed unchanged, as testified by the TGA curves recorded at T0 and T12, which are practically coincident (Fig. 11). A single-step degradation profile was observed with onset at ca. 275 °C and the maximum rate at ca. 290 °C, well in line with data reported for the thermal degradation of T-PHBV [33,34].

**Fig. 12:**
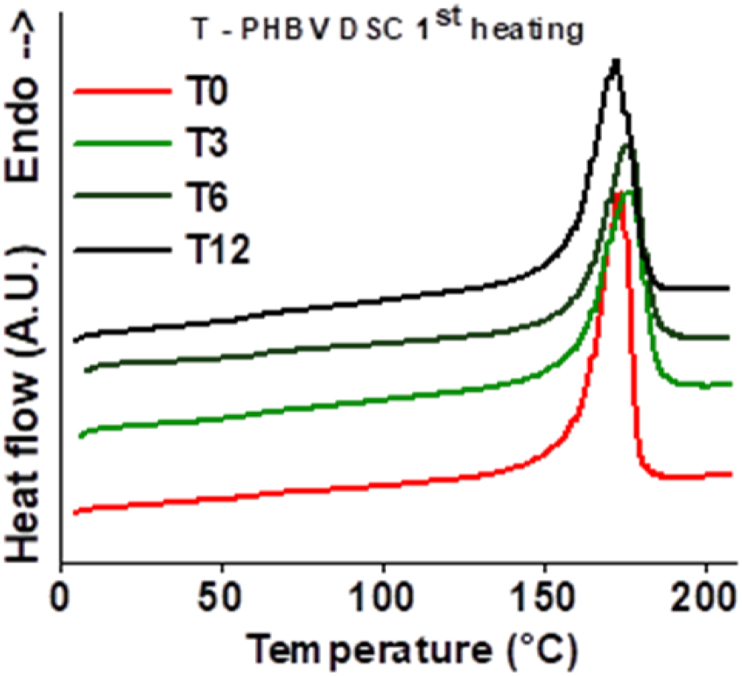
DSC 1st heating curves for T-PHBV at various times of exposure in Mediterranean seawater.

The DSC analysis (Fig. 12) evidenced that the transition temperatures (melting on first heating at 175 °C and crystallisation from subsequent cooling from the melt at 108 °C) remained practically equal to those of the unexposed samples upon exposure to a marine environment. The crystallinity degree, $X_{c}$ (%), was calculated (Equation (3)) measuring the melting enthalpy of the sample (${\Delta H}_{m}$) and considering as ${\Delta H}_{m}^{0}$ 146 J/g, i.e. the melting enthalpy of a theoretical 100 % crystalline poly(3-hydroxybutyrate) [33,35,36]. It fluctuates within 55–60 % over the whole seawater exposure period.

(3) $$X_{c}=\frac{{\Delta H}_{m}}{{\Delta H}_{m}^{0}\;}\times 100$$

By DSC no significant changes in melting temperature were observed for home composting and flow-through T-PHBV films, respectively. Since no changes in infrared spectra and thermal behaviour were observed during eight weeks in the home compost and 12 weeks in river Elbe, it indicated that T-PHBV degradation occurs at the surface and not in the polymer structure [37].

DSC of T-PHBV before and after the methane production potential test indicated that the peak temperature of crystallisation exotherm decreased from 102.8 °C to 100.3 °C and the related enthalpy decreased from −80 to −56 J/g. Furthermore, the peak point of melting temperature decreased from 167.0 °C to 163:8 °C and the related enthalpy decreased from 94 J/g to 64 J/g. It seems that the material reduced its degree of crystallinity from about 60 % to about 40 % (see Equation (3)) after 60 days of exposure to the above-mentioned conditions.

Crystallinity inhibits degradation as the permeation of enzymes and moisture are hindered by dense crystal areas. In case of PHA-copolymers, like PHBV, where HV-monomers have been linked into PHB, the crystallinity can be modified by HV/HB -ratio. The lower crystallinity is directly related to enzymatic degradation that has been proven both in aerobic and anaerobic circumstances [39]. DSC analysis showed a reduction of 20 %-units in crystallinity. Earlier, reduction in crystallinity was discovered in studies of Muniyasamy et al. [38], where PHBV was buried in soil, which can be considered as an anaerobic environment. The crystalline content of PHBV was changing during 30 days and 200 days incubation from 72,6 % to 74,3 %% and 26,6 % respectively. Thus, an increase in crystallinity was detected at first during the degradation. In the stydy, also significant reduction in melting temperature was observed from original 104,0 °C to 92,7 °C (30 days) and to 81,3 °C (200 days) [40]. In these studies of anaerobic degradation, more moderate shifts were noticed.

### 3.6 Morphological properties

**Fig. 13:**
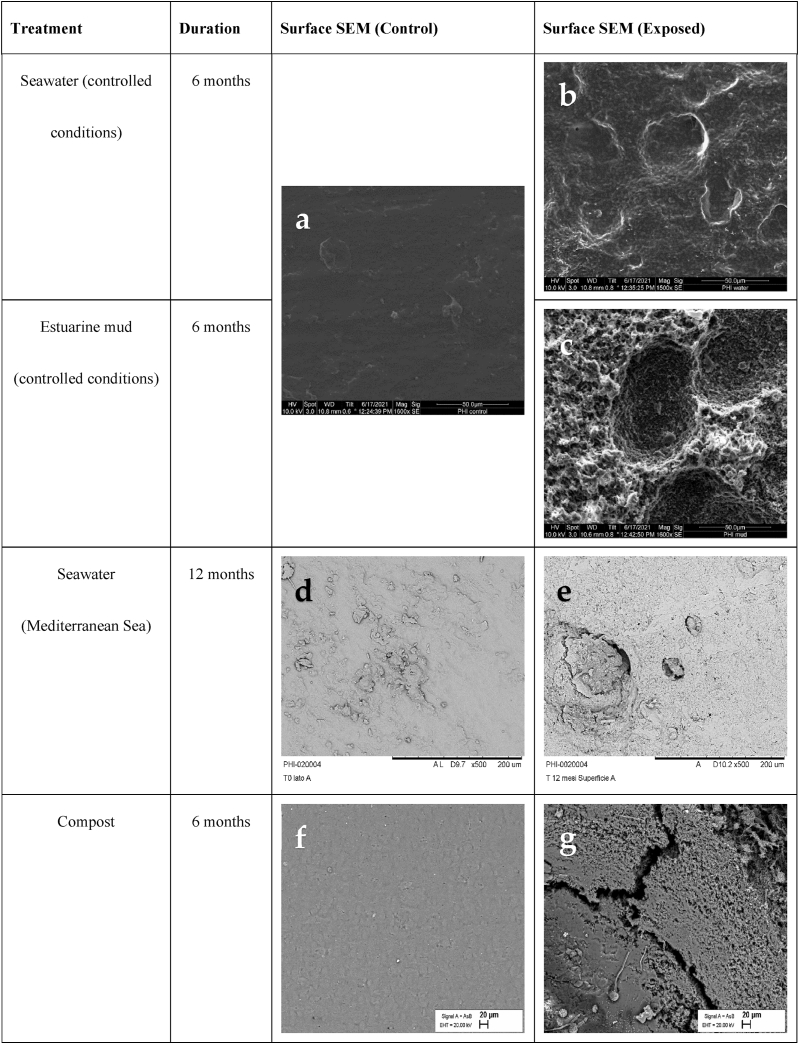

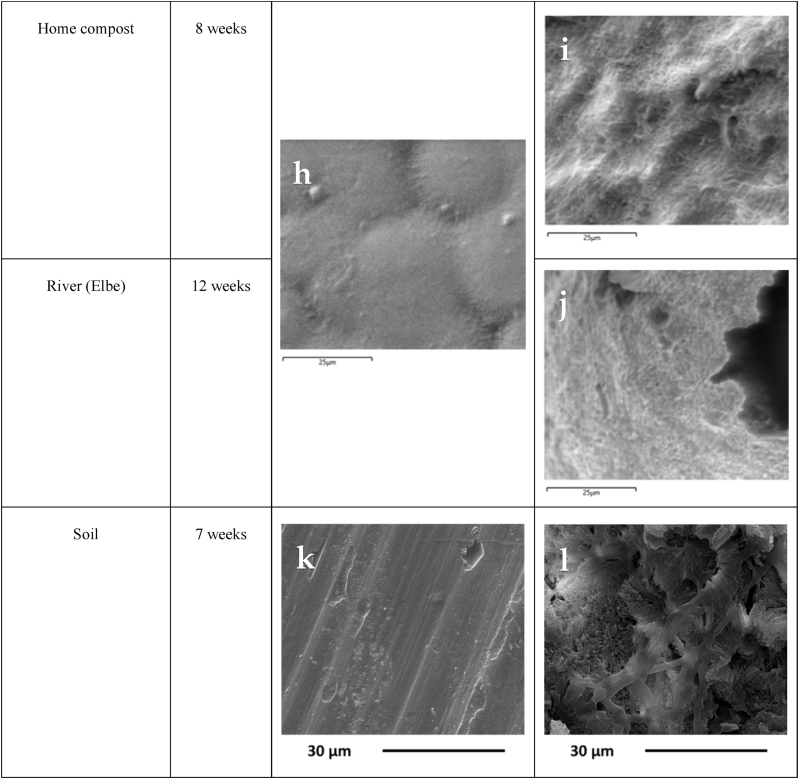
SEM pictures of the surfaces of untreated T-PHBV material (a, d, f, h, k) and after exposure to different conditions. b: Six months of exposure to seawater under controlled conditions. c: Six months exposure in estuarine mud under controlled conditions. e: Twelve months in Mediterranean seawater. g: Six months in compost. i: Eight weeks in home compost. j: Twelve weeks in a flow-through chamber in a river. l: Seven weeks in soil.

Morphological analysis by SEM was performed on the T-PHBV bars exposed to seawater and estuarine mud under controlled laboratory conditions. The incubated T-PHBV bars showed conspicuous erosion of the surface compared to the smooth surface of the pristine bars. After six months in seawater, the surface became rough and showed a multitude of depressions. After six months in estuarine mud, the erosion was even more pronounced. Large and deep pits covered the surface, indicating a gradual erosion of the top layer of the surface (Fig. 13a, b, c).

The scanning electron micrographs support the data of weight loss analysis. Degradation of the T-PHBV material in both seawater and estuarine mud is evident. As expected, all analyses indicate a stronger deterioration of the material in estuarine mud than in seawater. This stronger degradation in mud could be attributed to a higher prevalence of PHBV-degrading microorganisms and more favourable conditions for enzymatic activity. The shape of the depressions resembles those of microbes colonizing plastic surfaces retrieved from the subtropical North Atlantic [39]. The anaerobic conditions in the mud may have been a decisive factor, since many PHAs are better degraded in anoxic environments [40].

SEM micrographs of the T-PHBV bars exposed to Mediterranean seawater were taken at different magnications and several points of the exposed sample surface. A few micro defects or cracks observed on the exposed surface were already present on pristine samples (Fig. 13d). After 12 months, the exposed surface showed material erosion with the formation of sporadic voids of hundred microns and numerous micro holes of few-microns size, ascribed to slow material erosion (Fig. 13e same as abserved in Fig. 13d). Also, the two micrographs taken on T-PHBV sheets, before and after 12 months of immersion in the coastal North Sea water, evidence similar material erosion from the exposed sheet surface. SEM observations are in agreement with the ATR-FTIR analysis which indicate a certain extent of hydrolysis on the exosed sample surface.

Morphological analysis by SEM was performed on the T-PHBV granules during the composting process for 6 months. The original material showed a flat impermeable surface; however, after 6 months of composting, the surface became porous, and cracks appeared deep into the granule (Fig. 13f and g).

The occurrence of modifications after degradation experiments in home compost and exposition in the river Elbe was observed on the surface of T-PHBV films. Before incubation in home compost and exposition in the river Elbe, T-PHBV had a smooth surface with only a few cavities (Fig. 13h). The surface of T-PHBV films became very rough with various craters after eight weeks in a home compost (Figs. 13i) and 12 weeks in a flow-through chamber in river Elbe (Fig. 13j). Salomez et al. [11] observed an increase in porosity for T-PHBV material during composting, which is caused by microbial activity. In this study, the surface of T-PHBV film became more porous during home compost and freshwater degradation experiments, which indicated a good biodegradability of T-PHBV.

According to Vatanpour et al. [41], cellulose acetate can serve as a cost-effective additive to enhance various polymeric membranes due to its remarkable properties, such as hydrophilicity and high capacity for adsorbing dye molecules and heavy metals.

### 3.7 pH-stat

**Fig. 14:**
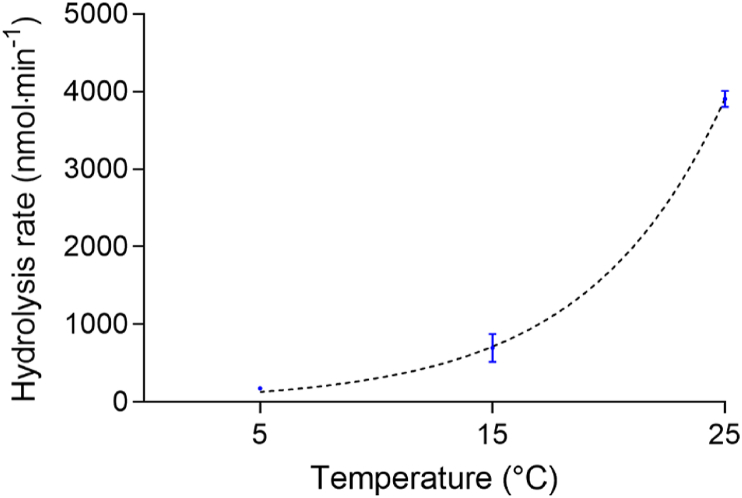
Hydrolytic degradation of T-PHBV by PHBV depolymerase measured by pH Stat titration at 5 °C, 15 °C, and 25 °C (means ± SD, n = 3).

T-PHBV-microparticles were hydrolysed by PHBV depolymerase. The hydrolysis rate increased exponentially with temperature to 3908 ± 108 nmol min^−1^ at 25 °C (Fig. 14). The exponential regression model explained 99 % of the temperature-induced variation of the hydrolysis rate.

The pH-Stat experiments proved an enzymatic degradability of the tested T-PHBV material by depolymerase, which is supported by several studies reporting enzymatic PHA degradation [[42], [43], [44]]. In nature, the environmental conditions in which the PHA material might end up often affect the rate of degradation. These comprise, besides others, oxygen availability, salinity, pH and temperature. With a strongly exponentially increasing hydrolysis rate of the T-PHBV material, our results confirm temperature as a crucial factor in the enzymatic degradation of T-PHBV. However, the temperature of natural seawater is mostly below 20 °C, reaching temperatures above 30 °C only in tropical surface waters [45]. Nonetheless, the rate of hydrolysis for T-PHBV at the lowest measured temperature (5 °C) is still quite high (179 ± 9 nmol min^−1^) when compared to other biodegradable plastics such as polylactic acid (0.5 ± 0.4 nmol min^−1^) or polybutylene succinate (1 ± 1 nmol min^−1^) [46]. When compared to the hydrolysis rates of the natural polymer collagen, measured by Miksch et al. [46] under similar conditions, T-PHBV seems to be even better degradable by depolymerase than collagen by protease (67.7 ± 5.7 nmol min^−1^). Depolymerase-producing microorganisms are present in a variety of marine habitats [47]. However, it is difficult to predict whether and to what extent depolymerases are active in such habitats. Although the *in-vitro* measurements show a high degradability of T-PHBV by depolymerase, the decomposition of the material in the exposition experiments does not match the high hydrolysis rate, indicating only a low depolymerase activity or low abundance of depolymerase-producing microorganisms under exposition conditions.

### 3.8 Evaluation of the degree of biodegradation under controlled aerobic conditions

**Fig. 15:**
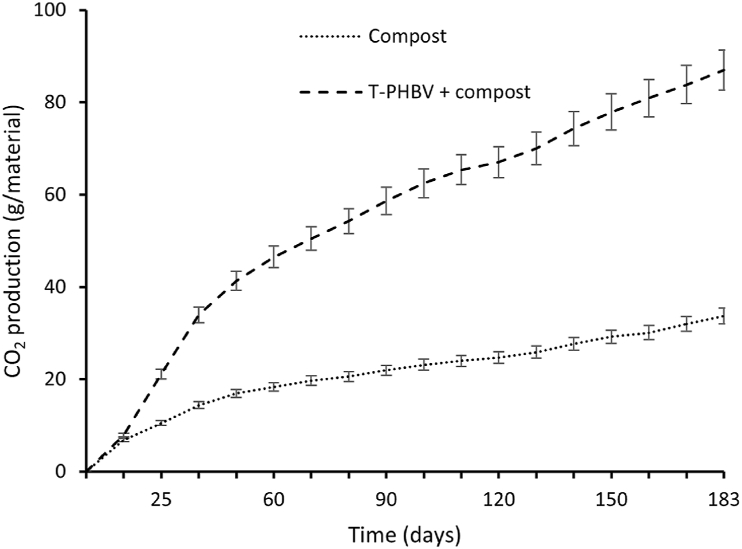
Carbon dioxide production monitored from compost only and compost mixed with T-PHBV monitored for 6 months.

For the 180 days of testing under controlled aerobic conditions, the mean value of carbon dioxide production for the vessels containing only compost reached 33.72 g, while the mean value for the vessels containing both compost and T-PHBV material reached 87 g (Fig. 15).

**Fig. 16:**
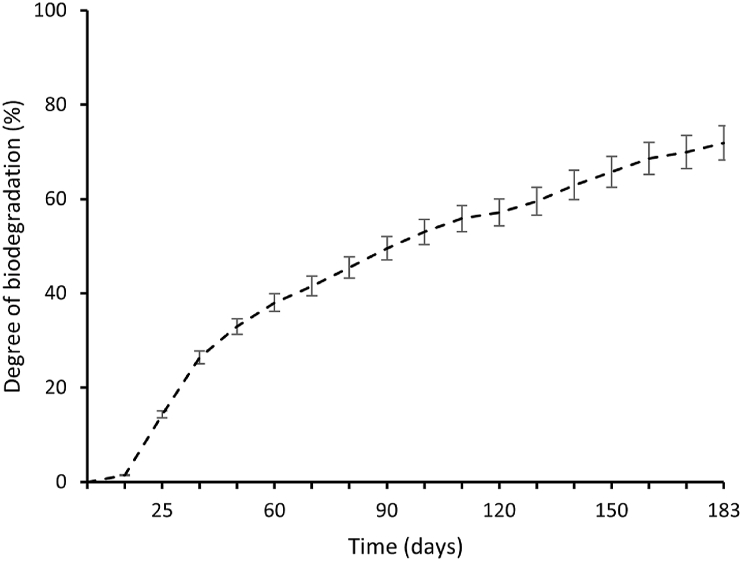
T-PHBV degree of biodegradation of the maximum degradation of TLC grade cellulose. Biodegradations were performed under controlled composting conditions from the beginning of the experiment until the end as described in Materials and Methods. The calculation was performed based on ISO 13432.

The degradation of T-PHBV started within 10 days, and previously no additional carbon dioxide was produced. After 25 days of testing, the carbon dioxide evolution increased and kept rising constantly to the 170th day and stabilised on the 180th day when the experiment was finished. By the end of the compostability testing, the T-PHBV material exhibited 71.9 % of biodegradation of the maximum degradation of TLC grade cellulose after a plateau was reached for both materials (Fig. 16).

Under comparable aerobic conditions, the material displayed a degradation rate of 53 % on the 100th day, while the pure PHBV material achieved 63.2 % degradation [48]. This suggests a reduction in the degree of degradation by 10.2 % under controlled composting conditions, as compared to the pure material.

### 3.9 Evaluation of methane produced in anaerobic conditions

**Fig. 17:**
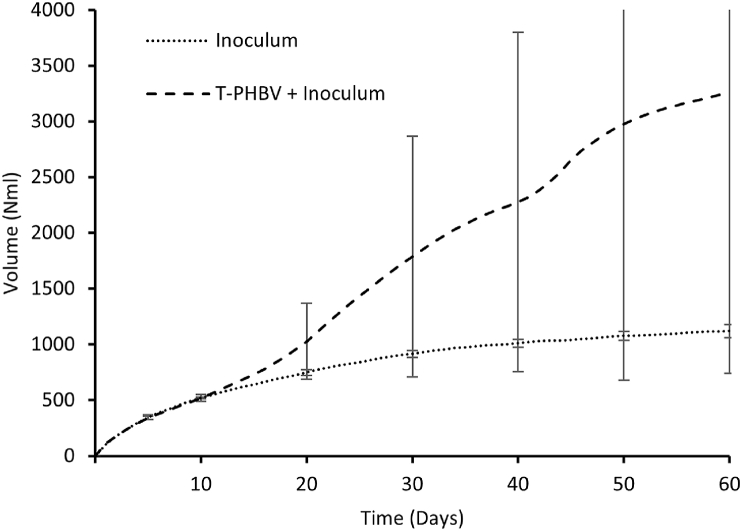
T-PHBV methane production potential test results for 60 days exposure.

During the 60-day methane potential test, the level of methane production of 0-samples (inoculum) was on average 1120 N ml and the level of methane production of T-PHBV samples (the mixtures of inoculum and the material) was on average 3260 N ml. It seems that the T-PHBV increased the methane production in the chamber; see Fig. 17. However, there was a significant difference between the T-PHBV samples in 2 parallel chambers (1480 Nml and 5040 Nml), while 2 0-samples showed uniform behaviour (1080 Nml and 1160 Nml). The methane production of the first T-PHBV chamber increased only 30 % when compared with the average of 0-sample chambers while the second T-PHBV chamber showed 350 % increase. The reason why the other T-PHBV sample degraded while the other did not is unknown. The inoculum was well mixed before splitting into different chambers, thus expected to be of homogenous quality. This expectation is also supported by the similar methane production behaviour of 0-samples. The different methane production potential of two arallel T-PHBV samples could be explained by differences in crystallinity because of heterogenic raw materials or differences in production parameters during moulding of the test bars [39]. However, no differences were noticed in pretests. Nevertheless, the difference in degradation between the 2 parallel samples can also be clearly detected visually (see Section 3.1).

Despite the promising results of sample 1 in methane production potential and acceptable degradation over 50 % in anaerobic conditions stated in standard EN13432, it must be noted that typically the retention time a biogas plant is 15–30 days, which based on these studies would be too short period for degradation of T-PHBV [49].

### 3.10 Total solids and volatile solids for plastic films in anaerobic conditions

Total solids and volatile solids express the amount of dry matter content (TS) and organic matter content (VS). The analysis of VS and TS was based on standard SFS-EN 15934, sludge, treated bio waste, soil and waste. Calculation of dry matter fraction after determination of dry residue or water content, where method A was used and standard SFS-EN 15935:2021 Soil, waste, treated bio waste and sludge. Determination of loss on ignition.

In the test, the sample of a known mass is heated to 105 °C for 22 h (overnight), weighed, and then further heated to 550 °C for 2 h and weighed again. The weight change is expressed in w% at 105 °C (TS) and 550 °C (VS).

The amount of total solids (TS) was 99.97 % before the 60 days exposure to anaerobic conditions and 99.08 % after the exposure. The amount of volatile solids (VS) before the exposure was 98.78 % and 97.92 % after the test. While the changes in TS and VS amounts seemed to be small they were statistically significant (p < 0,05). The VS/TS ratio before and after the exposure was 0.99. Thus, there were no changes in the VS/TS ratio. The result may be due to the relatively large size of the sample put in the chamber (Dumbbell 1A 150 mm × 4 mm test bar). It can be assumed that the degradation, typically to enzymatic degradation, occurred on the surface of the sample while the bulk has remained unchanged.

## 4 Conclusions

This paper has reviewed the degradation of the Poly(3-hydroxybutyrate-*co*-3-hydroxyvalerate) (PHBV) compound in different environments. PHBV is a thermoplastic bio-polyester produced from renewable vegetable resources, and it belongs to the polyhydroxyalkanoate (PHA) family.

The practical implications of this paper are two-fold. Firstly, the set of degradation tests performed on PHBV-based material showed that degradation of T-PHBV bars was found to be greater in natural estuarine mud compared to seawater. Secondly, during exposure, the edge of the film became discoloured, dark in colour after eight weeks and had cracks, which show that T-PHBV degradation occurred at the surface and not in the polymer structure.

In case of anaerobic degradation and methane production, noticeable differences were detected between the T-PHBV samples in 2 parallel chambers while 2 0-samples showed uniform behaviour. The first sample achieved acceptable level of degradation in anaerobic circumstances with potential for methane production while the second sample showed only moderate degradation.

The degradation is rather slow in the environment because it completely depends on the speed with which natural enzymes may act, UV resistance, low water absorption and different molecular sizes.

This study specifically aimed to investigate the biodegradability of PHBV under natural conditions, which implicates some limitations. First, natural conditions are always prone to seasonal variations (e.g. temperature, humidity) that are not fully controllable. Secondly, the study was not performed over a very long period of time (e.g. months to years), which limits the findings to the period during which the tests were performed. Nonetheless, our study provides important information on how PHBV degrades in different environments, how biodegradation proceeds over time and how this affects material properties.

The main strengths of the study lie in the fact it combines an assessment of the degradability of a PHBV-based compound under different conditions, using tools such as visual and mass changes, mechanical and morphological properties, spectroscopic and structural characterisation, along with thermal behaviour, methods which add a degree of robustness to the work undertaken.

In respect of future trends, it is very important that adequate measures are in place to better assist in the monitoring of the behaviour of PHBV in other types of environments. Also, further studies that investigate how PHBV interacts with other compounds are needed, in order to identify appropriate measures to optimise their use.

## Funding

Authors received funding from the European Union's 10.13039/501100007601Horizon 2020 research and innovation programme through the research project BIO-PLASTICS EUROPE, under grant agreement No. 860407.

## Data availability statement

Data will be made available on request.

## CRediT authorship contribution statement

**Pavlo Lyshtva:** Writing – review & editing, Writing – original draft, Methodology, Investigation, Formal analysis, Data curation, Conceptualization. **Viktoria Voronova:** Writing – original draft, Methodology. **Jelena Barbir:** Writing – review & editing, Writing – original draft. **Walter Leal Filho:** Writing – review & editing, Writing – original draft. **Silja Denise Kröger:** Writing – original draft, Methodology, Formal analysis. **Gesine Witt:** Writing – original draft, Methodology, Formal analysis. **Lukas Miksch:** Writing – original draft, Methodology, Formal analysis. **Reinhard Sabowski:** Writing – original draft, Methodology, Formal analysis. **Lars Gutow:** Writing – original draft, Methodology, Formal analysis. **Carina Frank:** Writing – original draft, Methodology, Formal analysis. **Anita Emmerstorfer-Augustin:** Writing – original draft, Methodology, Formal analysis. **Sarai Agustin-Salazar:** Writing – original draft, Methodology, Formal analysis. **Pierfrancesco Cerruti:** Writing – original draft, Methodology, Formal analysis. **Gabriella Santagata:** Writing – original draft, Methodology, Formal analysis. **Paola Stagnaro:** Writing – original draft, Methodology, Formal analysis. **Cristina D'Arrigo:** Writing – original draft, Methodology, Formal analysis. **Maurizio Vignolo:** Writing – original draft, Methodology, Formal analysis. **Anna-Sara Krång:** Writing – original draft, Methodology, Formal analysis. **Emma Strömberg:** Writing – original draft, Methodology, Formal analysis. **Liisa Lehtinen:** Writing – original draft, Methodology, Formal analysis. **Ville Annunen:** Writing – original draft, Methodology, Formal analysis.

## Declaration of competing interest

The authors declare that they have no known competing financial interests or personal relationships that could have appeared to influence the work reported in this paper.

The authors declare the following financial interests/personal relationships which may be considered as potential competing interests.

## References

[1] Thompson R.C., Swan S.H., Moore C.J., vom Saal F.S. (Jul. 2009). Our plastic age. Phil. Trans. R. Soc. B.

[2] Geyer R., Jambeck J.R., Law K.L. (Jul. 2017). Production, use, and fate of all plastics ever made. Sci. Adv..

[3] Patrício Silva A.L. (Feb. 2021). Increased plastic pollution due to COVID-19 pandemic: Challenges and recommendations. Chem. Eng. J..

[4] Plastics Europe Plastics – the facts 2022. https://plasticseurope.org/wp-content/uploads/2023/03/PE-PLASTICS-THE-FACTS_FINAL_DIGITAL-5.pdf.

[5] Weber M., Cocca M., Di Pace E., Errico M.E., Gentile G., Montarsolo A., Mossotti R. (2018). Proceedings of the International Conference on Microplastic Pollution in the Mediterranean Sea.

[6] Lammi S., Gastaldi E., Gaubiac F., Angellier-Coussy H. (2019). How olive pomace can be valorized as fillers to tune the biodegradation of PHBV based composites. Polym. Degrad. Stabil..

[7] Vermeer C.M. (Dec. 2022). Systematic solvent screening and selection for polyhydroxyalkanoates (PHBV) recovery from biomass. J. Environ. Chem. Eng..

[8] Pal A.K., Wu F., Misra M., Mohanty A.K. (Oct. 2020). Reactive extrusion of sustainable PHBV/PBAT-based nanocomposite films with organically modified nanoclay for packaging applications: compression moulding vs. cast film extrusion. Compos. B Eng..

[9] Mehrpouya M., Vahabi H., Barletta M., Laheurte P., Langlois V. (2021). Additive manufacturing of polyhydroxyalkanoates (PHAs) biopolymers: materials, printing techniques, and applications. Mater. Sci. Eng. C.

[10] Bonnenfant C., Gontard N., Aouf C. (2022). Biobased and biodegradable polymers in a circular economy context: understanding quercetin and gallic acid impacts on PHBV thermal properties. Polym. Degrad. Stabil..

[11] Salomez M. (Sep. 2019). A comparative study of degradation mechanisms of PHBV and PBSA under laboratory-scale composting conditions. Polym. Degrad. Stabil..

[12] Weng Y.-X., Wang Y., Wang X.-L., Wang Y.-Z. (Aug. 2010). Biodegradation behavior of PHBV films in a pilot-scale composting condition. Polym. Test..

[13] Kumar R., Sadeghi K., Jang J., Seo J. (Jul. 2023).

[14] Bellache R., Hammiche D., Bettache A., Boukerrou A. (2022). Enzymatic degradation of prickly pear seed (PPS)/Polyhydroxy(butyrate-co-valerate) (PHBV) biocomposite. Mater. Today: Proc..

[15] Numata K., Abe H., Doi Y. (Jun. 2008). Enzymatic processes for biodegradation of poly(hydroxyalkanoate)s crystals. Can. J. Chem..

[16] Sang B.I., Hori K., Tanji Y., Unno H. (2002). Fungal contribution to in situ biodegradation of poly(3-hydroxybutyrate-co-3-hydroxyvalerate) film in soil. Appl. Microbiol. Biotechnol..

[17] Rutkowska M. (Jul. 2008). Environmental degradation of blends of atactic poly[(R,S)-3-hydroxybutyrate] with natural PHBV in baltic Sea Water and compost with activated sludge. J. Polym. Environ..

[18] Luzier W.D. (Feb. 1992). Materials derived from biomass/biodegradable materials. Proc. Natl. Acad. Sci. U.S.A..

[19] Ashori A., Jonoobi M., Ayrilmis N., Shahreki A., Fashapoyeh M.A. (Sep. 2019). Preparation and characterization of polyhydroxybutyrate-co-valerate (PHBV) as green composites using nano reinforcements. Int. J. Biol. Macromol..

[20] Montanheiro T.L. do A. (Oct. 2019). Enhanced water uptake of PHBV scaffolds with functionalized cellulose nanocrystals. Polym. Test..

[21] Arrieta M.P., López J., Rayón E., Jiménez A. (Oct. 2014). Disintegrability under composting conditions of plasticized PLA–PHB blends. Polym. Degrad. Stabil..

[22] Miksch L., Gutow L., Saborowski R. (Mar. 2021). pH-stat titration: a rapid assay for enzymatic degradability of bio-based polymers. Polymers.

[23] Frank C., Emmerstorfer-Augustin A., Rath T., Trimmel G., Nachtnebel M., Stelzer F. (Jun. 2023). Bio-polyester/rubber compounds: fabrication, characterization, and biodegradation. Polymers.

[24] Meereboer K.W., Misra M., Mohanty A.K. (2020). Review of recent advances in the biodegradability of polyhydroxyalkanoate (PHA) bioplastics and their composites. Green Chem..

[25] Gonçalves S.P.C., Martins-Franchetti S.M., Chinaglia D.L. (Dec. 2009). Biodegradation of the films of PP, PHBV and its blend in soil. J. Polym. Environ..

[26] Zhu J. (Sep. 2019). Comprehensive insight into degradation mechanism of green biopolyester nanocomposites using functionalized cellulose nanocrystals. ACS Sustainable Chem. Eng..

[27] Zaidi Z., Mawad D., Crosky A. (2019). Soil biodegradation of unidirectional polyhydroxybutyrate-Co-valerate (PHBV) biocomposites toughened with polybutylene-adipate-Co-terephthalate (PBAT) and epoxidized natural rubber (ENR). Frontiers in Materials.

[28] Wu C.-S., Liao H.-T., Cai Y.-X. (2017). Characterisation, biodegradability and application of palm fibre-reinforced polyhydroxyalkanoate composites. Polym. Degrad. Stabil..

[29] Turco R., Santagata G., Corrado I., Pezzella C., Di Serio M. (2021). In vivo and post-synthesis strategies to enhance the properties of PHB-based materials: a review. Front. Bioeng. Biotechnol..

[30] Antunes A., Popelka A., Aljarod O., Hassan M.K., Kasak P., Luyt A.S. (2020). Accelerated weathering effects on poly(3-hydroxybutyrate-co-3-hydroxyvalerate) (PHBV) and PHBV/TiO2 nanocomposites. Polymers.

[31] Angelini S., Cerruti P., Immirzi B., Santagata G., Scarinzi G., Malinconico M. (Nov. 2014). From biowaste to bioresource: effect of a lignocellulosic filler on the properties of poly(3-hydroxybutyrate). Int. J. Biol. Macromol..

[32] Cai Y., Lv J., Feng J. (2013). Spectral characterization of four kinds of biodegradable plastics: poly (lactic acid), poly (butylenes adipate-Co-terephthalate), poly (hydroxybutyrate-Co-hydroxyvalerate) and poly (butylenes succinate) with FTIR and Raman spectroscopy. J. Polym. Environ..

[33] Li Z., Reimer C., Wang T., Mohanty A.K., Misra M. (2020). Thermal and Mechanical Properties of the Biocomposites of Miscanthus Biocarbon and Poly(3-Hydroxybutyrate-co-3-Hydroxyvalerate) (PHBV)’, Polymers.

[34] Seggiani M. (May 2018). Novel sustainable composites based on poly(hydroxybutyrate-co-hydroxyvalerate) and seagrass beach-CAST fibers: performance and degradability in marine environments. Materials.

[35] Barham P.J., Keller A., Otun E.L., Holmes P.A. (Sep. 1984). Crystallization and morphology of a bacterial thermoplastic: poly-3-hydroxybutyrate. J. Mater. Sci..

[36] Ten E., Jiang L., Wolcott M.P. (Sep. 2012). Crystallization kinetics of poly(3-hydroxybutyrate-co-3-hydroxyvalerate)/cellulose nanowhiskers composites. Carbohydr. Polym..

[37] Luo S., Netravali A.N. (2003). A study of physical and mechanical properties of poly(hydroxybutyrate-co-hydroxyvalerate) during composting. Polym. Degrad. Stabil..

[38] Muniyasamy S., Ofosu O., John M.J., Anandjiwala R.D. (Apr. 2016). Mineralization of poly(lactic acid) (PLA), poly(3-hydroxybutyrate-co-valerate) (PHBV) and PLA/PHBV blend in compost and soil environments. j renew mater.

[39] Zettler E.R., Mincer T.J., Amaral-Zettler L.A. (Jul. 2013). Life in the “plastisphere”: microbial communities on plastic marine debris. Environ. Sci. Technol..

[40] Abou-Zeid D.-M., Müller R.-J., Deckwer W.-D. (Mar. 2001). Degradation of natural and synthetic polyesters under anaerobic conditions. J. Biotechnol..

[41] Vatanpour V. (May 2022). Cellulose acetate in fabrication of polymeric membranes: a review. Chemosphere.

[42] Ghanem N.B., Mabrouk M.E.S., Sabry S.A., El-Badan D.E.S. (Jun. 2005). Degradation of polyesters by a novel marine Nocardiopsis aegyptia sp. nov.: application of Plackett-Burman experimental design for the improvement of PHB depolymerase activity. J. Gen. Appl. Microbiol..

[43] Kato C., Honma A., Sato S., Okura T., Fukuda R., Nogi Y. (2019). Poly 3-hydroxybutyrate-co-3-hydroxyhexanoate films can be degraded by the deep-sea microbes at high pressure and low temperature conditions. High Pres. Res..

[44] Zadjelovic V. (2020). Beyond oil degradation: enzymatic potential of Alcanivorax to degrade natural and synthetic polyesters: polyesters degradation by Alcanivorax. Environ. Microbiol..

[45] Deser C., Alexander M.A., Xie S.-P., Phillips A.S. (Jan. 2010). Sea surface temperature variability: patterns and mechanisms. Ann. Rev. Mar. Sci.

[46] Miksch L., Köck M., Gutow L., Saborowski R. (Jun. 2022). Bioplastics in the sea: rapid in-vitro evaluation of degradability and persistence at natural temperatures. Front. Mar. Sci..

[47] Suzuki M., Tachibana Y., Kasuya K. (Jan. 2021). Biodegradability of poly(3-hydroxyalkanoate) and poly(ε-caprolactone) via biological carbon cycles in marine environments. Polym. J..

[48] Muniyasamy S. (2019). Thermal-chemical and biodegradation behaviour of alginic acid treated flax fibres/poly(hydroxybutyrate-co-valerate) PHBV green composites in compost medium. Biocatal. Agric. Biotechnol..

[49] Bátori V., Åkesson D., Zamani A., Taherzadeh M.J., Sárvári Horváth I. (Oct. 2018). Anaerobic degradation of bioplastics: a review. Waste Manag..

